# Reactive Infiltration: Effects of Different Parameters

**DOI:** 10.3390/ma17133063

**Published:** 2024-06-21

**Authors:** M. Karla López-González, Leidy Figueroa-Quintero, David Villalgordo-Hernández, Enrique V. Ramos Fernández, Javier Narciso

**Affiliations:** 1 Laboratory of Advanced Materials, Inorganic Chemistry Department, University Materials Institute of Alicante, University of Alicante, 03080 Alicante, Spain; mariakarla.lopez@ua.es (M.K.L.-G.); leidy.figueroa11@ua.es (L.F.-Q.); david.villalgordo@ua.es (D.V.-H.); enrique.ramos@kaust.edu.sa (E.V.R.F.); 2 Alicante Institute for Health and Biomedical Research (ISABIAL), 03010 Alicante, Spain

**Keywords:** SiC, reactive infiltration, model

## Abstract

Currently, the production of complex SiC and SiC/SiC parts through reactive infiltration is one of the most widely used technologies, due to its versatility and cost-effectiveness compared to more conventional technologies such as Hot Isostatic Pressing (HIP). This technology, while widely adopted, still faces some debate regarding the mechanisms of infiltration. Questions persist about what determines how infiltration occurs and whether the process is governed by physics (flow dynamics) or chemistry (reactions at the triple line (LT: (contact line between the solid, liquid and gas phases)). The present work provides new strong/consistent proof that reactive infiltration is mainly controlled by chemical reaction.

## 1. Introduction

Si-based ceramics (SiC, Si_3_N_4_, SiAlON, …), also called non-oxide ceramics, are a group of materials with exceptional properties, among which SiC stands out as the prime example [1,2]. SiC is characterized by its high decomposition temperature, remarkable hardness, thermal conductivity surpassing any metal, and a moderate thermal coefficient of expansion [3,4,5]. Consequently, SiC has been used in a variety of applications, serving as a reinforcement in composite materials [4,5,6], in the electronics industry [7,8,9], and more recently in the manufacture of graphene [10,11], used in nuclear applications [12,13,14] and in the manufacture of furnace heating elements [15]. While the production of powder manufacturing, such as in monocrystalline whiskers, has been effectively addressed [16,17,18,19,20,21], the manufacture of pieces remains a challenge. 

Depending on the size and desired characteristics, monolithic SiC materials are mainly produced through three methods: sintering of SiC particles, reactive infiltration, or chemical vapor deposition (CVD) [2].

At the small centimeter scale, high-quality pieces can be obtained through Chemical Vapor Deposition (CVD) [22,23,24,25]. Through this process, it is possible to produce a solid form of SiC from a reactive gas-phase precursor at a comparatively low temperature compared to other methods (900–1100 °C).

Among the different methods for synthesizing SiC monoliths, sintering is one of the most commonly used. The most developed sintering processes are Hot Pressing (HP) and Hot Isostatic Pressing (HIP) [26,27,28,29]. The raw material used in both processes is very fine-grained SiC powder, previously synthesized by the Acheson process [1]. During sintering, densification is achieved by simultaneously heating and pressing the material in a mold with the desired shape. Industrially, temperatures between 1900 °C and 2300 °C are used, and materials are typically subjected to an extensive period of time to achieve high-quality materials (more than 2 h). The pressure strongly depends on the selected method; for HP processes, pressures between 10 MPa and 40 MPa are used, while for HIP processes, the pressure is in the range of 100 MPa to 400 MPa.

Given the extreme temperature and pressure conditions required for sintering, these processes are technically complex and are very costly from an economic standpoint.

The great revolution in manufacturing came from Popper [30], with the development of an alternative method for obtaining dense SiC materials known as reactive infiltration, also called RBSC (Reaction-bonded SiC) [31]. Later, the evolution of the microstructure was developed by Professor Page [32,33], who showed that two types of SiC are produced in association with the dissolution process of C in Si, and a subsequent precipitation of SiC, with a third associated with the epitaxial growth of the beta phase on the original SiC. The original idea was to join α-SiC with β-SiC [34]; for this purpose, α-SiC preforms were made with polymeric materials or pitches that, when treated at a high temperature, generated a porous network of carbon material between the carbide particles. These preforms were infiltrated with liquid silicon at temperatures between 1450 and 1600 °C because the infiltration is spontaneous, and during infiltration, the silicon react with carbon to form the new β-SiC that glues to the previous alpha SiC. It is not necessary to apply external pressure; normally, this process is carried out in an inert atmosphere or vacuum [35].

In general, the reactive infiltration method involves the infiltration of liquid silicon into a porous preform of carbon or carbon/α-SiC, where silicon reacts with carbon to form a dense SiC material [1,2]. This new manufacturing modality, in contrast to other SiC synthesis procedures, has proven to be more cost-effective from its inception because it uses lower temperatures than sintering (temperatures between 1450–1600 °C), does not require the application of external pressure (using an inert atmosphere or vacuum), and achieves synthesis of parts with complex geometries. For these reasons, the reactive infiltration method has established itself as one of the most attractive processes in the manufacturing of SiC materials [30], as well as C/SiC and SiC/SiC composite materials [34,35].

There are two variants of the infiltration method, which depend on the nature of the preform and determine the reaction that occurs in the process. In the case of preforms composed solely of carbon, the process is called RFSC (Reaction-formed SiC). In this process, silicon infiltrates the carbon preform, which produces an in situ reaction between these elements, forming β-SiC. In the second case, when the preform is composed of carbon and α-SiC, we have the RBSC method, as mentioned earlier. Here, the carbon that bonds the α-SiC particles transforms into β-SiC, forming a compound of α-SiC/β-SiC.

In recent years, the reactive infiltration process, has become increasingly popular; SiC/SiC parts are being manufactured using this technique for use in jet engines [36,37] and for brake discs in high-end cars [38,39]. However, there is still a great deal of controversy as to how the infiltration process occurs, with those from the school of engineering postulating that the process is controlled by fluid dynamics, meaning that the pores are closed and, therefore, infiltration is much slower [40,41,42,43]. However, the school of Professor Eustathopoulos proposes that it is controlled by the chemical reaction rate [44,45,46,47,48,49,50].

The present investigation is a continuation of previous research [51,52], with the aim of showing new evidence that the reactive infiltration process is controlled by chemical reaction and not by the fluid dynamics of the process.

## 2. Theoretical Background

The infiltration process in porous systems has been studied for more than 170 years, and some of these findings remain relevant today. Initially, the agricultural engineer Henry Darcy experimentally deduced the law that now bears his name [53], which was later deduced formally using the reduced Navier-Stokes equation [54], which is the formalism we will use in the present investigation.

The viscous flow of liquids is controlled by the Navier-Stokes equation. This differential equation is famously known to lack a general solution. However, under certain conditions, such as those present in this case, it is solvable by assuming an incompressible fluid in laminar flow (characterized by a low Reynolds number). Additionally, if the time derivative of the fluid velocity is much smaller than its spatial derivatives and the effects of gravity are considered negligible, the equation can be simplified to:

(1) $$\nabla p=\mu \nabla^{2}v,$$

where *v* is the speed of the fluid, *μ* is the dynamic viscosity, and *p* is the pressure. Assuming unidirectional flow (which is not so realistic in a standard porous system), it is possible to reduce the expression to Darcy’s law. This equation can be re-written in the most commonly used form to analyze the flow of incompressible fluids through porous media:

(2) $$\phi \frac{dz}{dt}=-\frac{K}{\mu }\frac{dp}{dz},$$

where *z* is the flow direction, *dp*/*dz* is the pressure gradient along the infiltration front, *ϕ* is the porosity, and *K* is the permeability of the porous media. It must be pointed out that the permeability of the porous media is proportional to the square of the average pore radius, meaning we can rewrite the equation where *a* is only the proportional constant:

(3) $$\frac{dz}{dt}=-a\frac{R^{2}}{\phi \mu }\frac{dp}{dz},$$

Since we are going to work with regular systems, we can use the Poiseuille equation in cylindrical coordinates where *r* is the radius, which takes this form:

(4) $$\frac{1}{r}\frac{\partial }{\partial r}\left(r\frac{\partial u}{\partial r}\right)=\frac{1}{\mu }\frac{dp}{dz},$$

Solving this, we can obtain the variation of the velocity, where *R* is the radius of the cylinder (pipe):

(5) $$u_{z}=-\frac{1}{4\mu }\left(R^{2}-r^{2}\right)\frac{dp}{dz},$$

Therefore, the maximum velocity would be:

(6) $$u_{zmax}=-\frac{R^{2}}{4\mu }\frac{dp}{dz},$$

Further, as it is well known, if the average velocity is 0.5 of the maximum velocity, the equation would be as follows:

(7) $$\frac{dz}{dt}=-\frac{R^{2}}{8\mu }\frac{dp}{dz},$$

which is the same as what we have previously deduced using Darcy’s law. Then, we make the following consideration:

(8) $$\frac{a}{\phi }=\frac{1}{8},$$

which tells us that regardless of the equation we solve, the evolution is the same—only one scalar varies. In order to relate the present investigation with the previous ones of our group, we will use the Darcy equation in its integrated form:

(9) $$h^{2}=\frac{2\cdot K\cdot t}{\mu \cdot \phi }\cdot ∆P$$

where *h* is the infiltrated distance. In the case of systems where the liquid wets the solid and infiltration proceeds spontaneously (contact angle, θ < 90°), the pressure drop is the capillary pressure (*P_c_*), given by:

(10) $$P_{c}=\frac{2\cdot \lambda \cdot \gamma_{LV}\cdot cos\theta \cdot (1-\Phi )}{r\cdot \phi }$$

where *λ* is a geometrical factor, *γ_LV_* is the surface tension of the liquid, *θ* is the contact angle of the liquid on the solid, *Φ* is the porosity of the system, and *r* is the average pore radius. Table 1 shows the parameters for the SiC systems used to calculate the infiltration time.

This model suggests that vertical infiltration of a preform of 5 mm thickness (penetration depth) should be achieved within a fraction of minute, 54 s. That is 2 orders of magnitude lower than what we observe experimentally [44,45,46,47,48,49,50,51,52,53].

Then, we can assume that the infiltration rate is much lower due to pore closure, and the closing of the pores is due to the fact that each C atom generates a SiC molecule, which causes an increase in volume of almost double, so we can reformulate the equation and assume that both radius and porosity are time-dependent, according to the following equation, assuming a cylindrical pore:

(11) $$\phi =\phi_{0}{\left(\frac{r}{r_{0}}\right)}^{2},$$

Taking this relationship into account, and the fact that the pressure will be the threshold pressure, we can obtain the following differential equation:

(12) $$dz^{2}=\frac{2a\lambda \gamma_{\mathrm{lv}}r_{0}^{2}\cos\theta }{\mu \phi_{0}r^{2}}\mathrm{drdt},$$

At this point, the only problem is to know the relationship between the radius and time. In the 1950s, the decreasing core model was proposed, one of the most widely used in chemical engineering, which could be controlled either by diffusion or by chemical reaction [61,62]. The equations derived are as follows:

- Diffusion

(13) $$t=\frac{M_{\mathrm{Si}}\rho_{\mathrm{SiC}}r_{0}^{2}}{6D_{C/\mathrm{SiC}}M_{\mathrm{SiC}}\rho_{\mathrm{Si}}}\left[1-3{\left(\frac{r}{r_{0}}\right)}^{2}+2{\left(\frac{r}{r_{0}}\right)}^{3}\right],$$

where *D_C_*_/*SiC*_ is the diffusion coefficient of C atoms in SiC, *M_Si_* and *M_SiC_* are the molecular mass of Si and SiC, respectively, and *ρ* is the density of both.

(14) $$\mathrm{dt}=\frac{M_{\mathrm{Si}}\rho_{\mathrm{SiC}}}{D_{C/\mathrm{SiC}}M_{\mathrm{SiC}}\rho_{\mathrm{Si}}}\cdot r\left[-1+\frac{r}{r_{0}}\right]\mathrm{dr},$$

- Chemical reactions

(15) $$t=\frac{M_{\mathrm{Si}}\rho_{\mathrm{SiC}}r_{0}}{K_{s}M_{\mathrm{SiC}}\rho_{\mathrm{Si}}}\left[1-\frac{r}{r_{0}}\right],$$

(16) $$\mathrm{dt}=-\frac{M_{\mathrm{Si}}\rho_{\mathrm{SiC}}}{K_{s}M_{\mathrm{SiC}}\rho_{\mathrm{Si}}}\mathrm{dr},$$

If we group the constants into a single term (*A* or *A*′), we would obtain the following equations to be solved:

(17) $$dz^{2}=A\;\left(-\frac{1}{r}+\frac{1}{r_{0}}\right)\mathrm{drdr},$$

(18) $$dz^{2}=A\prime \;\left(\frac{1}{r^{2}}\right)\mathrm{drdr},$$

Both equations are simple to solve with the appropriate boundary conditions, which, in any case, do not give linear equations, as observed experimentally [50]. Another simple way of looking at it is the time it would take to reduce the radius so that the velocity is of the order of that observed. To do this, we only have to substitute in the Equations (13) and (15) and we would obtain, in the first case, 2 h of diffusion time, and in the latter, a 1 h reaction time. In both cases, the rate of decrease of the pores is excessively small and does not fit the observed experimental data.

**Figure 1:**
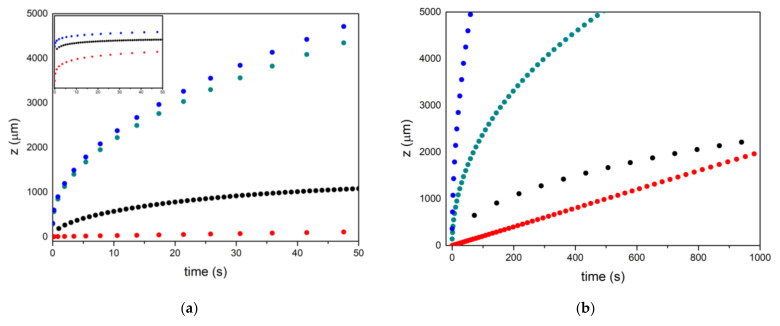
Evolution of infiltration as a function of time. Red is the experimental, blue is obtained by Darcy’s equation, green is the modified Darcy’s equation for radius reduction by silicon carbide formation controlled by Si diffusion in SiC, and black by the SiC formation reaction. (**a**) 30 micron pores; (**b**) 10 micron pores. Insert in (**a**) is the same graph in logarithmic scale.

Figure 1 shows the experimental data from [53], together with the simulations assuming that the decrease in velocity depends on the fluid dynamics and not on the chemical reaction. In the case where the decrease is controlled by diffusion, it clearly shows that there is very little variation (compare the black and green dotted lines), although a considerable decrease is observed in the case where it is controlled by the chemical reaction rate (see the black dotted line). It can therefore be inferred that for pores with a radius of the order of 20–30 microns, the infiltration rate will depend on the growth rate of SiC in the triple lines [54]. For smaller pore sizes of the order of 5–10 microns, it would be controlled by the SiC formation reaction rate of wall growth, since both models predict a very similar evolution of the pore size, especially after 1000 s (compare the red and black dotted lines), as shown in the simulation in Figure 1b for a radius of 5 microns.

**Figure 2:**
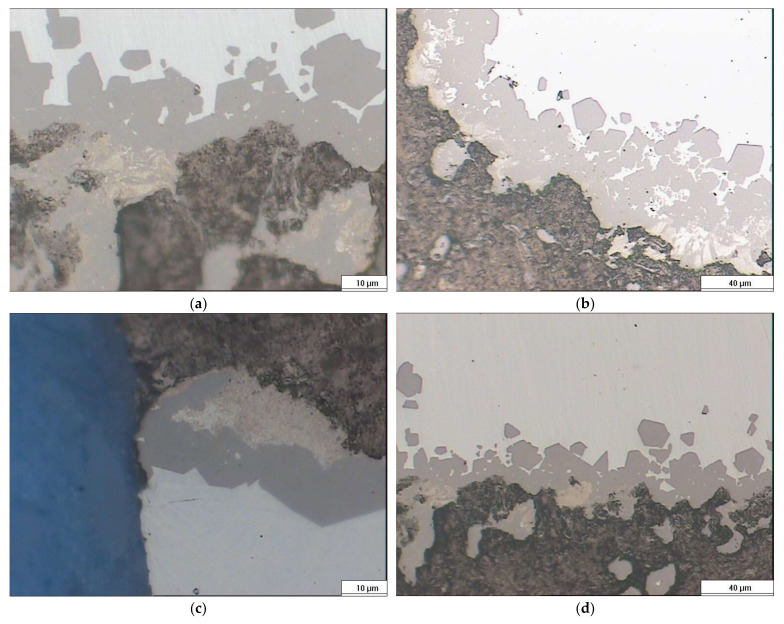
Micrographs of infiltrated capillary at different augments. Carbon is the black zone, the dark grey zone is the faceted SiC, the light brown is the “pocket” SiC, and the “white” zone is the not-reacted Si.

The phenomenology of SiC growth is rather more complex, as it has been described that the process occurs at least in two stages. In the first one, the formation of perfectly well-defined crystals of the order of 2–5 microns occurs, but they do not form a continuum, leaving the infiltration of silicon between them, which reacts with C to form a series of smaller SiC, which would form a continuum, and from here on, the growth would be controlled by diffusion, see Figure 2a–d.

More recent works carried out with capillaries [63,64], in which a detailed study of all the variables such as temperature and vacuum level was carried out, showing similar results to those we previously obtained [51,52]. In all cases, they showed an absolute linearity between infiltration and time, and the activation energy was found to be 280 KJ/mol, very similar to that found by Eustathopoulos et al. [49,50], which was the second major argument to show that infiltration is controlled by chemical reaction. Other experiments recently carried out by Ortona et al. [65,66,67,68] again highlight the importance of the reaction in the reactive infiltration process, in this case, even working with much more complex systems (alloys, creeping capillaries).

## 3. Experimental

**Figure 3:**
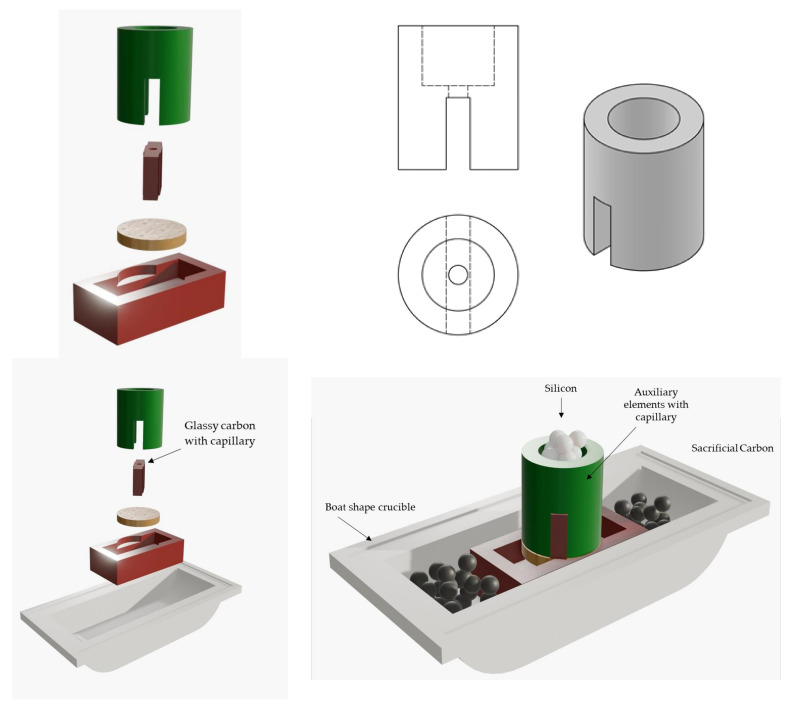
The setup engineered for the experiments of reactive infiltration.

The experiment of [55] has been reproduced. The difference is that the Si evolution is now followed in an equivalent way to the experiments carried out to determine the surface tension [46]. The experimental design scheme is shown in Figure 3.

The experiments are carried out on two types of carbon materials, glassy carbon (supplied by Goodfellow, Huntingdon, UK) on the one hand, and high-density synthetic graphite (supplied by Schunck, Munich, Germany) on the other. The characteristics are given in [48] and the silicon used is of electronic quality (supplied by Goodfellow). Precise control over the infiltration process is attained by utilizing boat-shaped alumina crucibles for assembling the components. High-density alumina is often selected for high-temperature applications due to its thermal, mechanical, and chemical durability. Auxiliary elements consist of graphite block coated with boron nitride to prevent unwanted reactions with molten silicon. To counteract adverse effects of residual oxygen, the sample is encased in activated carbon particles smaller than 1 mm. All experiments are carried out at 1450 °C in a vacuum of 10^−4^ mbar, using a vacuum system (membrane pump + turbomolecular pump, model HiCUBE supplied by Pfeiffer Vacuum, Asslar, Germany). The channel diameter is 500 × 10^−6^ m, using mechanical methods (drilling). At the end of the experiment, the sample was sectioned and polished according to the standard methods of [50]. The sections were analyzed by Scanning Electron Microscopy (SEM) and the analysis was performed by using a S3000N microscope (Hitachi, Tokyo, Japan) and optical microscopy (Leica MC170 HD, Wetzlar, Germany).

## 4. Results and Discussion

The objective of this study is to understand which phenomena govern the infiltration of SiC/C preforms, defining whether the infiltration is governed by chemical reaction or fluid dynamic phenomena. A study of the infiltration kinetics was conducted by analyzing the evolution of the droplet volume (V) as a function of time.

It is considered that the molten droplet during infiltration maintains a spherical cap geometry. This fact, combined with knowledge of geometric variables (such as the contact angle (θ), base radius (R), and height (h)), enables the calculation of the droplet volume (V) at each instant. This calculation is summarized in Equation (19), which describes the volume of a spherical cap as a function of the droplet height (h) and base radius (R_B_):

(19) $$V=\frac{\pi h}{6}(3{R_{B}}^{2}+h^{2}),$$

Additionally, knowing the initial volume of the droplet (*V*_0_), it is possible to determine the volume of liquid infiltrated at any given moment (*V_Inf_*), calculated as:

(20) $$V_{inf}=V_{0}-V,$$

Initially, the sections from various experiments were analyzed, confirming the earlier observations: well-formed silicon carbide, trapped silicon, and silicon carbide of a significantly smaller size, in the range of 100–200 nm (refer to Figure 2, brown zone).

**Figure 4:**
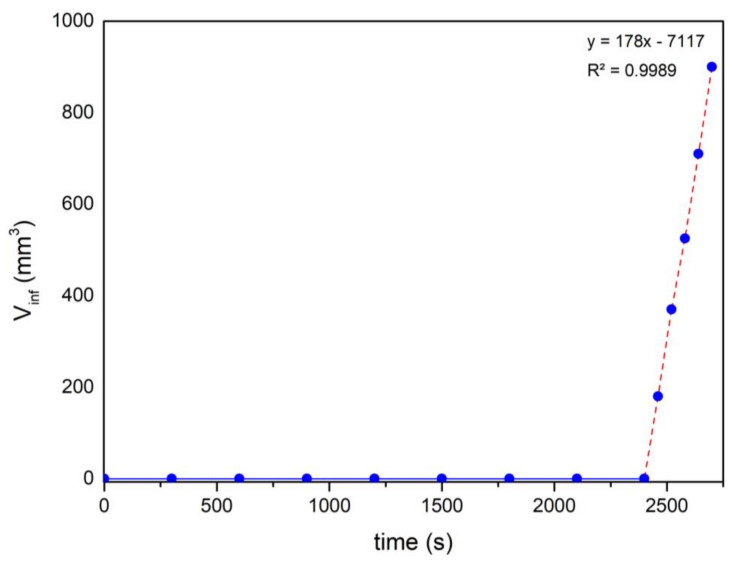
Evolution of infiltrated volume at function of time.

The most interesting results are shown in Figure 4, which shows the evolution of the infiltrated volume (*V_Inf_*) with time (t). During the first 40 min, no change is observed; this is the time of penetration of Si through the capillary, which tells us that the infiltration velocity is of the order of 2 to 2.1 μm/s, which is very similar to that which has been previously reported [51]. From that moment on, we see a constant increment of the infiltration volume, which, in principle, would tell us that what is happening is a flow of a liquid through a pipe. To analyse the data, the boundary conditions must be established and the pressure exerted must be calculated.

(21) $$\mathrm{Capillarity}\;P=\frac{2\gamma_{\mathrm{lv}}\cos\theta }{r}=\frac{2\times \;0.75\;\cos(40)}{250\;\times \;10^{-6}}=4500\mathrm{Pa},$$

(22) Hydrostatic P=ρgh=2570×9.8×0.02=500 Pa,

For capillaries larger than 500 μm in diameter, the hydrostatic pressure is no longer negligible, so the pressure is 5000 Pa. Then, to calculate the flow rate through the capillary, we apply the Poiseuille equation:

(23) $$Q=\frac{∆P\pi r^{4}}{8\;\mu L}=\frac{5000\times 3.14159\;{\left(250\times 10^{-6}\right)}^{4}}{8\times 6.05\times 10^{-2}\times 0.005}=2.54\times 10^{-8}\frac{m^{3}}{s}=25.4\frac{{\mathrm{mm}}^{3}}{s}$$

The flow rate obtained is an order of magnitude less than 3 mm^3^/s, as can be inferred from Figure 4.

No difference has been observed between the two materials. These experiments corroborate those mentioned above; the infiltration rate is controlled by the chemical reaction in the triple line. As soon as the “porous” zone is covered with SiC, the infiltration proceeds according to the well-known laws of fluid flow.

## 5. Conclusions

In the present research, it has been investigated, both from a theoretical and experimental point of view, which factors govern the Si infiltration process of a porous carbon preform. From a theoretical point of view, the equations have been formulated assuming that the infiltration process is controlled by fluid dynamics and that the experimentally low infiltration rate is due to the formation of SiC on the walls, which decreases the pore size. In no case has it been found that the infiltration rate is linear and that the rate is similar to that measured experimentally in preforms with an average pore size of 50 microns. An extrapolation has been carried out for smaller diameters, finding that it could partially explain the infiltration in only one case, and only as long as the pore is less than 5 microns.

Experimentally for a 500-micron cylindric pore, the data show that SiC formation occurs in several stages, and that once a continuous layer is formed, infiltration proceeds according to the classical Poiseuille law. This is only possible if the infiltration rate in the first stage is controlled by the reaction rate in the triple line.

## Figures and Tables

**Table 1:** Parameters used for the calculation of the infiltration time of liquid Si on the different preforms at 1450 °C.

Symbol	Magnitude	Value	SI Units	References
*λ*	Geometrical factor	2	-	[52,55,56]
*a*	Proportionality constant	4 × 10^−4^	-	[57]
*γ_LV_*	Surface tension	0.750	N/m	[58,59,60]
θ	Contact angle of Si on C	40	°	[45]
μ	Viscosity of liquid Si	6.05 × 10^−2^	Pa·s	[58]

## Data Availability

The data presented in this study are available in the article.

## References

[1] Calderon N.R., Martinez-Escandell M., Narciso J., Rodríguez-Reinoso F. (2009). The role of carbon biotemplate density in mechanical properties of biomorphic SiC. J. Eur. Ceram. Soc..

[2] Calderon N.R., Martinez-Escandell M., Narciso J., Rodríguez-Reinoso F. (2010). Manufacture of Biomorphic SiC Components with Homogeneous Properties from Sawdust by Reactive Infiltration with Liquid Silicon. J. Am. Ceram. Soc..

[3] Wu R., Zhou K., Yue C.Y., Wei J., Pan Y. (2015). Recent progress in synthesis, properties and potential applications of SiC nanomaterials. Prog. Mater. Sci..

[4] Choyke W.J., Pensl G. (1997). Physical Properties of SiC. MRS Bull..

[5] Mortensen A., Llorca J. (2010). Metal Matrix Composites. Annu. Rev. Mater. Res..

[6] Mussatto A., Ahad I.U., Mousavian R.T., Delaure Y., Brabazon D. (2021). Advanced production routes for metal matrix composites. Eng. Rep..

[7] Ozpineci B., Tolbert L.M. (2003). Characterization of SiC Schottky Diodes at Different Temperatures. IEEE Power Electron. Lett..

[8] Rupp R., Treu M., Voss S., Bjork F., Reimann T. “2nd Generation” SiC Schottky diodes: A new benchmark in SiC device ruggedness. Proceedings of the 18th International Symposium on Power Semiconductor Devices & IC’s.

[9] Neudeck P.G. (1995). Progress in silicon carbide semiconductor electronics technology. J. Electron. Mater..

[10] Lara-Avila S., Danilov A., Golubev D., He H., Kim K.H., Yakimova R., Lombardi F., Bauch T., Cherednichenko S., Kubatkin S. (2019). Towards quantum-limited coherent detection of terahertz waves in charge-neutral graphene. Nat. Astron..

[11] Cacccia M., Molina-Jordá J.M., Moral M., Nowak R., Ricci E., Sobczak N., Narciso J., Fernández-Sanz J. (2018). Graphene Translucency and Interfacial Interactions in the Gold/Graphene/SiC System. J. Phys. Chem. Lett..

[12] Katoh Y., Snead L.L., Szlufarska I., Weber W.J. (2012). Radiation effects in SiC for nuclear structural applications. Curr. Opin. Solid State Mater. Sci..

[13] Koyanagi T., Katoh Y., Nozawa T., Snead L.L., Kondo S., Henager C.H., Ferraris M., Hinoki T., Huang Q. (2018). Recent progress in the development of SiC composites for nuclear fusion applications. J. Nucl. Mater..

[14] Katoh Y., Ozawa K., Shih C., Nozawa T., Shinavski R.J., Hasegawa A., Snead L.L. (2014). Continuous SiC fiber, CVI SiC matrix composites for nuclear applications: Properties and irradiation effects. J. Nucl. Mater..

[15] Gnesin G.G., Dyban Y.P., Osovitskii E.J. (1975). Development of a high-density silicon carbide material for heating elements using a planning method. Powder Metall. Met. Ceram..

[16] Ramesh P.D., Vaidhyanathan B., Ganguli M., Rao K.J. (1994). Synthesis of β-SiC powder by use of microwave radiation. J. Mater. Res..

[17] Li X., Zhang G., Tronstad R., Ostrvski O. (2016). Synthesis of SiC whiskers by VLS and VS process. Ceram. Int..

[18] Chiew Y.L., Cheong K.Y. (2011). A review on the synthesis of SiC from plant-based biomasses. Mater. Sci. Eng. B.

[19] El-Eskandarany M.S., Sumiyama K., Suzuki K. (1995). Mechanical solid state reaction for synthesis of β—Sic powders. J. Mater. Res..

[20] Chaira D., Mishra B.K., Sangal S. (2008). Synthesis and characterization of silicon carbide by reaction milling in a dual-drive planetary mill. Mater. Sci. Eng. A.

[21] Chavda M.A., Ojovan M.I., Zhang S. Immobilization of Nuclear Waste Graphite Using the SiC Synthesis Route. Proceedings of the WM’11 Conference.

[22] Ellison A., Zhang J., Peterson J., Henry A., Wahab Q., Bergman J.P., Makarov Y.N., Vorob’ev A., Vehanen A., Janzén E. (1999). High temperature CVD growth of SiC. Mater. Sci. Eng. B.

[23] Kordina O., Hallin C., Henry A., Bergman J.P., Ivanov I., Ellison A., Son N.T., Janzén E. (1997). Growth of SiC by “Hot-Wall” CVD and HTCVD. PSS B.

[24] Pedersen H., Leone S., Kordina O., Henry A., Nishizawa S., Koshka Y., Janzén E. (2012). Chloride-Based CVD Growth of Silicon Carbide for Electronic Applications. Chem. Rev..

[25] Kuo D.H., Cheng D.J., Shyy W.J., Hon M.H. (1990). The Effect of CH 4 on CVD β-SiC Growth. J. Electrochem. Soc..

[26] Shi J., Guo J., Jiang D. (1993). Hot isostatic pressing of α-silicon carbide ceramics. Ceram. Int..

[27] Watson G.K., Moore T.J., Millard M.L. (1985). Effect of hot isostatic pressing on the properties of sintered alpha silicon carbide. Am. Ceram. Soc. Bull..

[28] Lange F.F. (1975). Hot-pressing behaviour of silicon carbide powders with additions of aluminium oxide. J. Mater. Sci..

[29] Singhal S.C., Lange F.F. (1975). Effect of Alumina Content on the Oxidation of Hot-Pressed Silicon Carbide. J. Am. Ceram. Soc..

[30] Popper P., Davies D.G.S. (1961). The preparation and properties of self-bonded silicon carbide. Powder Met..

[31] Aroati S., Cafri M., Dilman H., Dariel M.P., Frage N. (2011). Preparation of reaction bonded silicon carbide (RBSC) using boron carbide as an alternative source of carbón. J. Eur. Ceram. Soc..

[32] Jepps N.W., Page T.F. (1983). Polytypic transformations in silicon carbide. Prog. Cryst. Growth Charact..

[33] Ness J.N., Page T.F. (1986). Microstructural evolution in reaction-bonded silicon carbide. J. Mater. Sci..

[34] Ramos-Fernández E., Narciso J. (2023). Manufacture of SiC: Effect of Carbon Precursor. Materials.

[35] Caccia M., Narciso J. (2019). Key Parameters in the Manufacture of SiC-Based Composite Materials by Reactive Melt Infiltration. Materials.

[36] Naslain R., Christin F. (2003). Ultrahigh-Temperature Materials for Jet Engines. MRS Bull..

[37] Ohnabe H., Masaki S., Onozuka M., Miyahara K., Sasa T. (1999). Potential application of ceramic matrix composites to aero-engine components. Compos. Part A Appl. Sci. Manuf..

[38] Krenkel W., Heidenreich B., Renz R. (2002). C/C-SiC Composites for Advanced Friction Systems. Adv. Eng. Mater..

[39] Krenkel W., Berndt F. (2005). C/C–SiC composites for space applications and advanced friction systems. Mater. Sci. Eng. A.

[40] Einset E.O. (1996). Capillary Infiltration Rates into Porous Media with Applications to Silcomp Processing. J. Am. Ceram. Soc..

[41] Einset E.O. (1998). Analysis of reactive melt infiltration in the processing of ceramics and ceramic composites. Chem. Eng. Sci..

[42] Roger J., Chollon G. (2019). Mechanisms and kinetics during reactive infiltration of molten silicon in porous graphite. Ceram. Int..

[43] Sangsuwan P., Tewari S.N., Gatica J.E., Singh M., Dickerson R. (1999). Reactive infiltration of silicon melt through microporous amorphous carbon preforms. Met. Mater. Trans. B.

[44] Dezellus O., Jacques S., Hodaj F., Eustathopoulos N. (2005). Wetting and infiltration of carbon by liquid silicon. J. Mater. Sci..

[45] Eustathopoulos N., Nicholas M.G., Drevet B. (1999). Wettability at High Temperatures: Volume 3 (Pergamon Materials Series).

[46] Eustathopoulos N. (2005). Progress in understanding and modeling reactive wetting of metals on ceramics. Curr. Opin. Solid State Mater. Sci..

[47] Landry K., Rado C., Voitivich R., Eustathopulos N. (1997). Mechanisms of reactive wetting: The question of triple line configuration. Acta Mater..

[48] Caccia M., Amore S., Giuranno D., Novakovic R., Ricci E., Narciso J. (2015). Towards optimization of SiC/CoSi2 composite material manufacture via reactive infiltration: Wetting study of Si–Co alloys on carbon materials. Eur. Ceram. Soc..

[49] Calderon N.R., Voytovych R., Narciso J., Eustathopoulos N. (2010). Pressureless infiltration versus wetting in AlSi/graphite system. J. Mater. Sci..

[50] Bougiouri V., Voytovych R., Rojo-Calderon N., Narciso J., Eustathopoulos N. (2006). The role of the chemical reaction in the infiltration of porous carbon by NiSi alloys. Scr. Mater..

[51] Sergi D., Camarano A., Molina J.M., Ortona A., Narciso J. (2016). Surface growth for molten silicon infiltration into carbon millimeter-sized channels: Lattice–Boltzmann simulations, experiments and models. Int. J. Mod. Phys. C.

[52] Caccia M.R., Camarano A., Sergi D., Ortona A., Narciso J., Aliofkhazraei M. (2015). Wetting and Navier-Stokes Equation—The Manufacture of Composites Materials. Wetting and Wettability.

[53] Darcy H., Dalmont V. (1856). Les Fontaines Publiques de la Ville de Dijon: Exposition et Application des Principes a Suivre et des Formulesa Employer dans les Questions de Distribution d’Eau.

[54] Whitaker S. (1986). Flow in porous media I: A theoretical derivation of Darcy’s law. Transp. Porous Media.

[55] Garcia-Cordovilla C., Louis E., Narciso J. (1999). Pressure infiltration of packed ceramic particulates by liquid metals. Acta Mater..

[56] Alonso A., Pamies A., Narciso J., Garcia-Cordovilla C., Louis E. (1993). Evaluation of the wettability of liquid aluminum with ceramic particulates (SiC, TiC, AI_2_O_3_) by means of pressure infiltration. Metall. Trans. A.

[57] De Arellano-Lopez A.R., Martinez-Fernandez J.J., Gonzalez P., Dominguez C., Fernandez-Quero V., Singh M. (2004). Biomorphic SiC: A new engineering ceramic material. Appl. Ceram. Technol..

[58] Amore S., Giuranno D., Novakovic R., Ricci E., Nowak R., Sobczak N. (2014). Thermodynamic and surface properties of liquid Ge-Si alloys. Calphad.

[59] Novakovic R., Ricci E., Gnecco F., Giuranno D., Borzone G. (2005). Surface and transport properties of Au-Sn liquid alloys. Surf. Sci..

[60] Egry I., Ricci E., Novakovic R., Ozawa S. (2010). Surface tension of liquid metals and alloys—Recent developments. Adv. Colloid Interface Sci..

[61] Levenspiel O. (2006). Chemical Reaction Engineering.

[62] Levenspiel O. (2010). Chemical Reaction Omnibook.

[63] Hofbauer P. (2019). In-Situ Measurement and Simulation of the Liquid Silicon Infiltration Process. Ph.D. Thesis.

[64] Hofbauer P.J., Rädlein E., Raether F. (2018). Fundamental Mechanisms with Reactive Infiltration of Silicon Melt Into Carbon Capillaries. Adv. Eng. Mater..

[65] Naikade M., Hain C., Kastelik K., Parrilli A., Graule T., Weber L., Ortona A. (2023). Liquid metal infiltration of silicon based alloys into porous carbonaceous materials Part-III: Experimental verification of conversion products and infiltration depth by infiltration of Si-Zr alloy into mixed SiC/graphite preforms. J. Eur. Ceram. Soc..

[66] Naikade M., Ortona A., Graule T., Weber L. (2022). Liquid metal infiltration of silicon based alloys into porous carbonaceous materials. Part I: Modelling of channel filling and reaction phase formation. J. Eur. Ceram. Soc..

[67] Naikade M., Hain C., Kastelik K., Brönnimann R., Bianchi G., Ortona A., Graule T., Weber L. (2022). Liquid metal infiltration of silicon based alloys into porous carbonaceous materials. Part II: Experimental verification of modelling approaches by infiltration of Si-Zr alloy into idealized microchannels. J. Eur. Ceram. Soc..

[68] Sergi D., Grossi L., Leidi T. (2016). Simulation of capillary infiltration into packing structures for the optimization of ceramic materials using the lattice Boltzmann method. Eng. Appl. Comput. Fluid Mech..

